# Pathway Dependence of the Formation and Development of Prefibrillar Aggregates in Insulin B Chain

**DOI:** 10.3390/molecules27133964

**Published:** 2022-06-21

**Authors:** Yuki Yoshikawa, Keisuke Yuzu, Naoki Yamamoto, Ken Morishima, Rintaro Inoue, Masaaki Sugiyama, Tetsushi Iwasaki, Masatomo So, Yuji Goto, Atsuo Tamura, Eri Chatani

**Affiliations:** 1 Graduate School of Science, Kobe University, 1-1 Rokkodai, Nada, Kobe 657-8501, Hyogo, Japan; yoshikawa.yuki@kao.com (Y.Y.); 192s227s@stu.kobe-u.ac.jp (K.Y.); tiwasaki@kobe-u.ac.jp (T.I.); tamuatsu@kobe-u.ac.jp (A.T.); 2 Division of Biophysics, Physiology, School of Medicine, Jichi Medical University, 3311-1 Yakushiji, Shimotsuke 329-0498, Tochigi, Japan; nyamamoto@jichi.ac.jp; 3 Institute for Integrated Radiation and Nuclear Science, Kyoto University, 2 Asashiro-Nishi, Kumatori, Sennan-gun 590-0494, Osaka, Japan; morishima.ken.8e@kyoto-u.ac.jp (K.M.); inoue.rintaro.5w@kyoto-u.ac.jp (R.I.); sugiyama.masaaki.5n@kyoto-u.ac.jp (M.S.); 4 Biosignal Research Center, Kobe University, 1-1 Rokkodai, Nada, Kobe 657-8501, Hyogo, Japan; 5 Institute for Protein Research, Osaka University, 3-2 Yamadaoka, Suita 565-0871, Osaka, Japan; m.so@leeds.ac.uk; 6 Global Center for Medical Engineering and Informatics, Osaka University, 2-1 Yamadaoka, Suita 565-0871, Osaka, Japan; gtyj8126@protein.osaka-u.ac.jp

**Keywords:** amyloid, insulin B chain, nucleation, prefibrillar aggregates, protofibrils

## Abstract

Amyloid fibrils have been an important subject as they are involved in the development of many amyloidoses and neurodegenerative diseases. The formation of amyloid fibrils is typically initiated by nucleation, whereas its exact mechanisms are largely unknown. With this situation, we have previously identified prefibrillar aggregates in the formation of insulin B chain amyloid fibrils, which have provided an insight into the mechanisms of protein assembly involved in nucleation. Here, we have investigated the formation of insulin B chain amyloid fibrils under different pH conditions to better understand amyloid nucleation mediated by prefibrillar aggregates. The B chain showed strong propensity to form amyloid fibrils over a wide pH range, and prefibrillar aggregates were formed under all examined conditions. In particular, different structures of amyloid fibrils were found at pH 5.2 and pH 8.7, making it possible to compare different pathways. Detailed investigations at pH 5.2 in comparison with those at pH 8.7 have suggested that the evolution of protofibril-like aggregates is a common mechanism. In addition, different processes of evolution of the prefibrillar aggregates have also been identified, suggesting that the nucleation processes diversify depending on the polymorphism of amyloid fibrils.

## 1. Introduction

Amyloid fibrils are protein aggregates that are associated with many serious human diseases [1]. They typically show needle-like morphology, which is formed by the intertwining of protofilaments with a characteristic cross-β structure [2,3,4]. Recently, detailed structural investigations using cryo-electron microscopy and solid-state NMR techniques have determined many structures of amyloid fibrils at higher spatial resolution [5,6,7]. The molecular structures have demonstrated a common characteristic that polypeptide chains are folded into a planar structure, and then stack along the fibril axis. This is suggestive of highly ordered and periodic architecture of amyloid fibrils, and, probably because of this structure property, the formation of amyloid fibrils typically exhibits a nucleation step similar to the crystallization of a wide range of substances [8,9]. Nucleation is the first step in the emergence of amyloid structures in a reaction system, and primarily determines the progression of amyloid formation as well as the length of the lag phase as a rate-limiting step. Although recent modeling analyses have suggested that secondary nucleation and fragmentation are also involved in the late lag phase [10,11,12], investigating early aggregation of protein or peptide molecules is important for elucidating the initiation of amyloid formation and for developing strategies for preventing and treating amyloid-related diseases at early stages. However, detailed mechanisms of protein assembly that progresses during the formation of amyloid nuclei remain largely unclear.

To elucidate the mechanism of amyloid nucleation, studies have been carried out to propose appropriate reaction schemes as well as kinetic parameters that can reproduce experimental observations. The most fundamental and simplest mechanism of nucleation is assumed to progress in one step, and in this case, the process is accomplished without passing through any thermodynamically stable intermediates [13]. On the other hand, prefibrillar aggregates have often been identified in early stages of amyloid formation reactions, and some of them have been suggested to play an important role for amyloid nucleation [14,15,16,17]. The nucleus conformational conversion (NCC) proposed in the Sup35 study is the first model describing multistep nucleation mediated by prefibrillar aggregates [14], and since then, the involvement of oligomers in nucleation has often been argued. Nevertheless, previous studies have identified aggregates involved as off-pathway intermediates in the formation of amyloid fibrils [18,19], and the role of prefibrillar aggregates often seems controversial. Trapping and characterizing structures and formation processes of prefibrillar aggregates in a wide range of proteins and discussing their detailed roles in nucleation are, therefore, important for facilitating our understanding of protein assembly mechanisms in the initial stage of amyloid formation.

Given this research background, we previously found that prefibrillar aggregates function as a nucleation intermediate in the amyloid formation of an insulin-derived peptide B chain. Insulin is composed of two polypeptide chains, and the B chain is one of them, composed of 30 amino acid residues. High amyloid propensity of the B chain was previously revealed [20], and in our analysis, large amounts of prefibrillar aggregates were clarified to accumulate before the appearance of amyloid fibrils [21]. The formed prefibrillar aggregates were metastable and the application of agitation significantly promoted the formation of amyloid fibrils. It was also suggested that targeting the prefibrillar aggregates is an efficient strategy to inhibit amyloid formation [22]. These observations indicate the deep involvement of the B chain prefibrillar aggregates in the nucleation process. It is therefore expected that the B chain will serve as a useful model system for examining the detailed mechanism of the nucleation process mediated by prefibrillar aggregates.

In this study, we have found that similar prefibrillar aggregate-mediated amyloid formation proceeds over a wide pH range between 5.2 and 9.1. With a main focus on the amyloid formation at pH 5.2 showing a different pathway from that previously investigated at pH 8.7 [21,22], we have analyzed structural properties of prefibrillar aggregates and their formation processes through the combination of various analytical techniques, i.e., thioflavin T (ThT) fluorescence, attenuated total reflectance Fourier transform infrared (ATR-FTIR) spectroscopy, circular dichroism (CD) spectroscopy, atomic force microscopy (AFM), small-angle X-ray scattering (SAXS), and dynamic light scattering (DLS). The structural properties of the prefibrillar aggregates have suggested that they are protofibril-like, exhibiting partially organized β-structure and rod-like shape. It has also been clarified that both of their structure and size gradually developed during the formation of prefibrillar aggregates. By measuring the time course of prefibrillar aggregation of the B chain at pH 5.2, we discuss the formation processes of prefibrillar aggregates, and furthermore, their pathway dependence in comparison with that at pH 8.7.

## 2. Results

### 2.1. Amyloid Formation at Various pH Values

**Figure 1:**
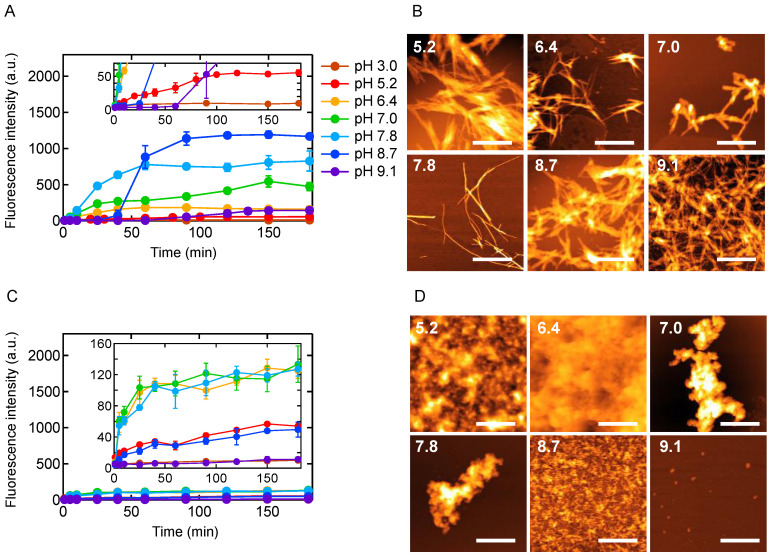
Aggregation reactions of insulin B chain at various pHs under (**A**,**B**) agitated and (**C**,**D**) quiescent conditions. The B chain at a concentration of 1.40 mg/mL was incubated at 25 °C, and in agitated conditions, the sample was shaken continuously at 1200 rpm. (**A**,**C**) Time courses of ThT fluorescence intensity under (**A**) agitated or (**C**) quiescent conditions. The mean values of triple data are plotted with standard deviations as error bars. (**B**,**D**) AFM images of aggregates after incubation for 2.5 h under (**B**) agitated or (**D**) quiescent conditions. Scale bars represent 1 μm.

We monitored aggregation reactions at different pH values ranging from 3.0 to 9.1. When reactions were performed under agitated conditions (i.e., shaking at 1200 rpm), an increase in ThT fluorescence intensity was observed at almost all pH conditions except pH 3.0 (Figure 1A). In addition, the AFM images of the reaction products with positive ThT fluorescence intensity showed characteristic morphologies typical of amyloid fibrils (Figure 1B), suggesting that the B chain has a strong propensity to form amyloid fibrils over a wide pH range. At pH 5.2, many amyloid fibrils were observed in the AFM image despite the fact that the ThT fluorescence intensity was lower than the others (Figure 1A, inset, and Figure 1B).

In our previous investigation at pH 8.7, prefibrillar aggregates were transiently accumulated early in the amyloid formation reaction [21]. They were characterized as a metastable species being trapped under quiescent conditions. To examine whether similar prefibrillar aggregates are involved at different pH values other than pH 8.7, aggregation reactions were monitored without agitation. As a result, a slight increase in ThT fluorescence was observed at pH range between 5.2 and 8.7 (Figure 1C). When the morphology of the reaction products was analyzed by AFM, nonfibrillar aggregates were observed in all conditions showing positive ThT fluorescence (Figure 1D). At pH 9.1, a small number of particle-like aggregates was observed even though ThT fluorescence was almost under detection limits (Figure 1C, inset, and Figure 1D), suggesting that nonfibrillar aggregation progresses to some extent. Taken together, amyloid formation accompanying nonfibrillar aggregates has been suggested to progress under a wide range of pH between 5.2 and 9.1.

### 2.2. Structure of Nonfibrillar Aggregates Formed under Quiescent Conditions

**Figure 2:**
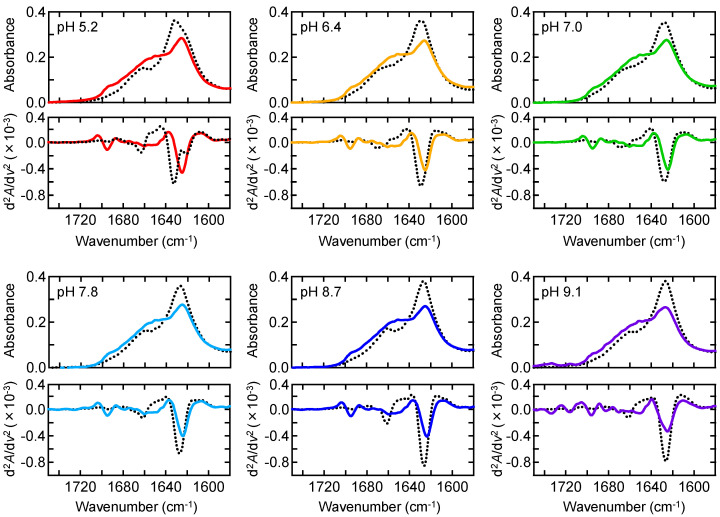
Structural properties of nonfibrillar aggregates formed under quiescent conditions at various pHs. ATR-FTIR absorption spectra and their second derivatives are shown in upper and lower panels, respectively. Solid lines indicate the spectra of nonfibrillar aggregates formed under quiescent conditions, and the spectra of amyloid fibrils formed under agitated conditions are also represented by black dotted lines. Spectra were normalized so that the integrated intensity of the amide I band ranging from 1580 to 1750 cm^−1^ was set to be equal.

To characterize the structure of nonfibrillar aggregates observed under quiescent conditions, we measured ATR-FTIR spectra. Although the amount of nonfibrillar aggregates formed at pH 9.1 was small, the measurement was realized by increasing sample volume. At all pH values, the aggregates were richer in random coil, as represented by a larger peak around 1650 cm^−1^, than in amyloid fibrils, although the β-sheet component at around 1625 cm^−1^ was contained to some extent (Figure 2). This suggests that nonfibrillar aggregates are not fully disordered but contain β structure. The β-sheet peak of nonfibrillar aggregates tended to be located slightly at a lower wavenumber than that of amyloid fibrils. Furthermore, a characteristic peak was commonly observed at around 1695 cm^−1^, presumably corresponding to antiparallel β-sheet [23]. These spectral properties suggest that the β structure contained in nonfibrillar aggregates has different properties from that of amyloid fibrils.

**Figure 3:**
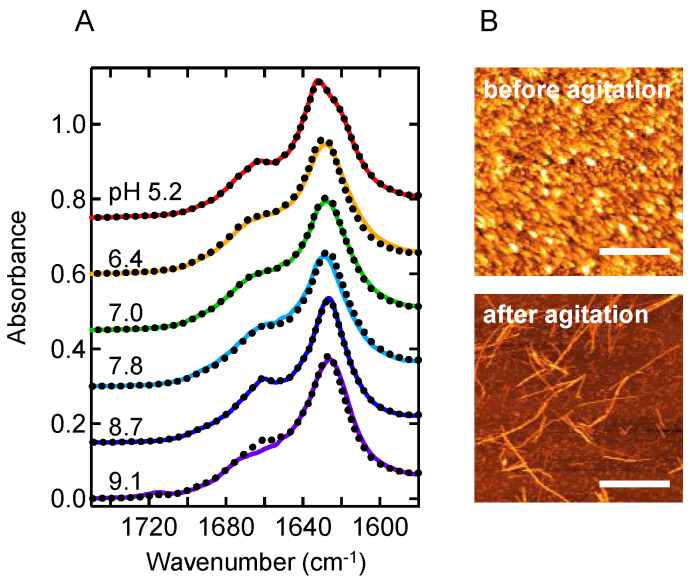
Agitation-induced conversion of nonfibrillar aggregates to amyloid fibrils. (**A**) ATR-FTIR absorption spectra of nonfibrillar aggregates measured after subjected to agitation. The spectra of amyloid fibrils formed under agitated conditions (the same spectra shown in Figure 2) are overlaid with dotted lines for references. (**B**) AFM images at pH 5.2 before and after agitation. Scale bars represent 1 μm.

We next examined whether the nonfibrillar aggregates play a role as intermediates of amyloid formation. In the previous work conducted at pH 8.7, nonfibrillar aggregates formed under quiescent conditions were transformed into amyloid fibrils by supplying a mechanical stimulus with shaking or ultrasonic wave [21]. To test whether similar agitation-induced conversion to amyloid fibrils occurs at other pH conditions, nonfibrillar aggregates formed by incubating for 2.5 h under quiescent conditions were shaken for 2.5 h, and then FTIR spectra were measured. The resulting spectra showed a similar shape to that of amyloid fibrils in all conditions (Figure 3A), and the formation of amyloid fibrils was confirmed by AFM at pH 5.2 as a representative (Figure 3B), raising a possibility that these nonfibrillar aggregates function as precursors of amyloid fibrils.

### 2.3. Formation Process of Prefibrillar Aggregates at pH 5.2

When the FTIR spectra of the formed amyloid fibrils were compared at different pHs, the position and intensity of main absorption peaks showed pH dependence. In particular, the spectrum at pH 5.2 had two β-sheet peaks at 1621 cm^−1^ and 1632 cm^−1^, which were clearly identified by the second derivative, suggesting that the pH 5.2 amyloid fibrils contain two types of β-sheet structures with different hydrogen bond arrangements. On the other hand, the spectra at the other pH values showed no clear separation of the β-sheet peak (Figure 2). The difference in amyloid structure between pH 5.2 and pH 8.7 was also supported by a comparison of proteinase K digestion (Figure S1) and cytotoxicity (Figure S2). Given the polymorphism in the final product, it was expected that the formation of B chain amyloid fibrils proceeds at pH 5.2 in a different pathway from other pH conditions. We therefore selected pH 5.2 as a target of investigation in comparison with pH 8.7 analyzed in the previous study [21,22].

**Figure 4:**
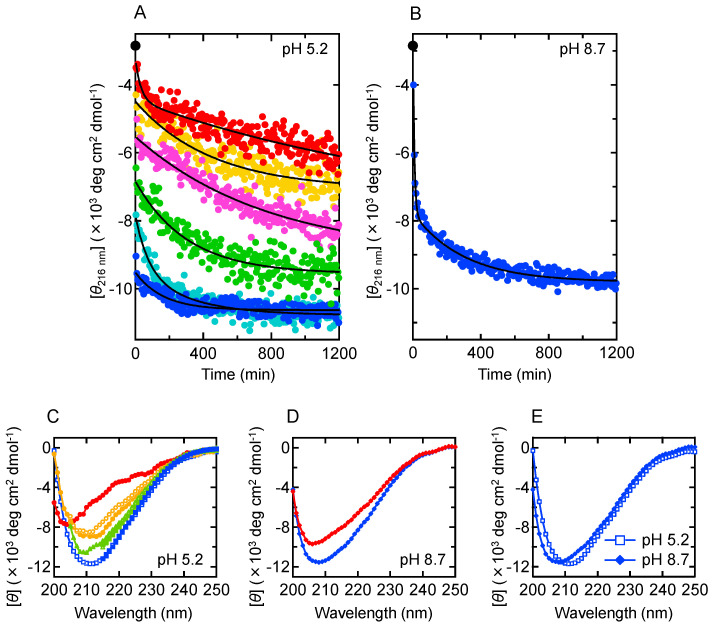
Difference in the formation process of prefibrillar aggregates between pH 5.2 and pH 8.7 as revealed by CD spectral changes. (**A**) Time-dependent changes in the values of mean residue molar ellipticity at 216 nm [*θ*_216 nm_] at pH 5.2. Plots at six different peptide concentrations (0.22 mg/mL, red; 0.25 mg/mL, orange; 0.30 mg/mL, magenta; 0.50 mg/mL, green; 0.70 mg/mL, cyan; 1.40 mg/mL, blue) are shown. Black lines represent regression curves obtained by fitting analysis with an exponential or a biexponential function, using Equation (1) (for 0.25 mg/mL, 0.30 mg/mL, 0.50 mg/mL, and 1.40 mg/mL) or Equation (2) (at 0.22 mg/mL and 0.70 mg/mL). A black circle indicates the value of the monomeric B chain measured in NaOH. (**B**) Time-dependent changes in the values of [*θ*_216 nm_] at pH 8.7. Plots at a peptide concentration of 1.40 mg/mL are shown. A black line represents a regression curve obtained by spectral fitting using a biexponential function, Equation (2). A black circle indicates the value of the monomeric B chain. (**C**) CD spectra of intermediate states at pH 5.2 reproduced by spectral extrapolation at each phase in the spectral fitting. Overlaying spectra obtained at different peptide concentrations (closed circles, 0.22 mg/mL; open circles, 0.25 mg/mL; closed triangles, 0.30 mg/mL, open triangles, 0.50 mg/mL; closed squares, 0.70 mg/mL; open squares, 1.40 mg/mL) suggested four different states, which are colored by red, orange, green, and blue in order of appearance, respectively. (**D**) CD spectra of intermediate states at pH 8.7 reproduced by spectral extrapolation at each phase in the spectral fitting. The result suggested two different states, as shown by red and blue diamonds in order of appearance. (**E**) Comparison of final convergent spectra between the pH 5.2 and the pH 8.7 prefibrillar aggregates.

To characterize the detailed process of aggregation at pH 5.2, a time-dependent change in secondary structure was tracked by using far-UV CD spectra. The spectrum changed in two steps under agitated conditions, supporting that the formation of amyloid fibrils progresses via prefibrillar aggregates (Figure S3). The formation process of prefibrillar aggregates was successfully observed under quiescent conditions, although it was much faster than at pH 8.7, showing a large burst phase (Figure 4A,B, blue plots). When additional measurements were performed at lower B chain concentrations, earlier structural changes could be captured because aggregation rate slowed down. The time-dependent changes in spectra were reasonably explained by an exponential or a biexponential function at each B chain concentration (Figure 4A). Combining all the convergent spectra estimated by the fitting of the spectral time courses, we found that the change in CD spectrum converged to a total of four different spectra, i.e., the first and second spectra at 0.22 mg/mL; the second spectrum at 0.25 mg/mL; the third spectrum at 0.30 mg/mL; the third spectrum at 0.50 mg/mL; the third and fourth spectra at 0.70 mg/mL; and the fourth spectrum at 1.40 mg/mL (Figure 4C). Given that each of them was observed for at least two different concentrations, the appearance of these spectra was considered to be sequential over the increase of peptide concentration. This result suggests that there are four steps in secondary structural change during the formation of prefibrillar aggregates at pH 5.2. At pH 8.7, on the other hand, the spectral change at 1.40 mg/mL did not show a significant burst phase, and two steps were identified (Figure 4D). The difference in the number of steps suggests that the process of prefibrillar aggregation diversifies in a pathway-dependent manner. In addition, although difficult to detect in FTIR spectroscopy, the final shape of the CD spectrum showed a slightly different minimum wavelength between pH 5.2 and 8.7 (Figure 4E), implying that these prefibrillar aggregates have structural differences to some extent.

**Figure 5:**
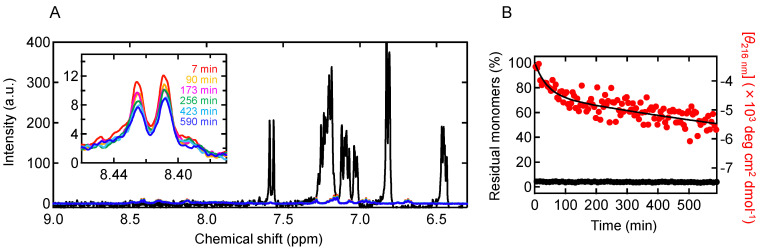
Quantification of residual monomers in the process of the formation of prefibrillar aggregates at pH 5.2 using ^1^H-NMR spectra. (**A**) Time-dependent change in ^1^H-NMR spectra in a low magnetic field region. The measurement was performed at a B chain concentration of 0.22 mg/mL. Inset shows a magnified view of peaks of two histidine ε protons, which were used for quantifying the fraction of residual monomers. The black line is the spectrum of a monomer state obtained with the B chain dissolved in a NaOH solution, and it should be noted that the peaks of two histidine ε protons are observed at around 7.6 ppm due to difference in pH. (**B**) Time course of the fraction of the residual monomers estimated from the area of the histidine peaks. For comparison, the time-dependent change in [*θ*_216 nm_] at 0.22 mg/mL shown in Figure 4A is overlaid.

To track the progress of aggregation during the organization of secondary structure, the time course measurement of ^1^H-NMR spectra was also performed. It was conducted at a peptide concentration of 0.22 mg/mL, the lowest concentration used for the CD measurement to track the earliest conformational changes (see Figure 4A). The results showed that proton signals almost disappeared immediately after the start of the reaction, suggesting rapid progression of aggregation (Figure 5A). The decay of the NMR peaks demonstrated that the monomer concentration dropped to 4% within 7 min from the start of the reaction, and by comparing with the changes in CD spectra, it was revealed that disordered aggregation occurred before the formation of secondary structure (Figure 5B). Considering that the proton signal typically disappears when the molecular diameter becomes 30–40 nm [21], the initial aggregates were estimated to have a significant number of associations of the B chain peptides. However, their accurate size could not be measured by DLS or SAXS in this work due to the experimental limitation that their formation was only allowed at low peptide concentrations. This reaction process is obviously different when compared to the reaction at pH 8.7, where aggregation and conformational changes occur synchronously [21]. 

### 2.4. Size and Shape of the pH 5.2 Aggregates

**Figure 6:**
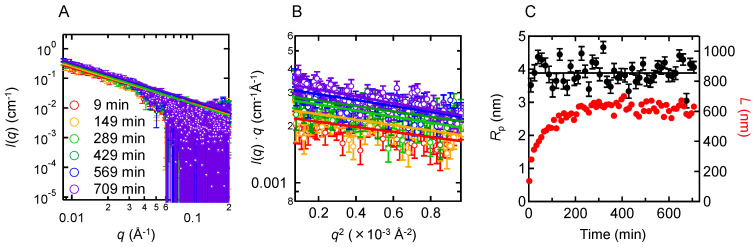
Characterization of the size and shape of prefibrillar aggregates at pH 5.2 monitored by SAXS. The measurement was performed at 1.40 mg/mL. (**A**) Double logarithm plots of the SAXS profiles at different time points. Solid lines indicate fitted lines using Equation (3) in Porod region. (**B**) Cross-section plots. Solid lines indicate fitted curves obtained using Equation (4). (**C**) Time changes of *R*_p_ (black) and *L* (red), which were obtained from *R*_c_ in panel B and the DLS data in Figure S4 using Equations (5) and (6). A solid line indicates the mean of the *R*_p_ values.

To analyze the size and shape of prefibrillar aggregates formed at pH 5.2, SAXS measurements were performed. The B chain solution at pH 5.2 was loaded into the cell at a concentration of 1.40 mg/mL, and SAXS profiles were obtained continuously until the reaction completed. Figure 6A shows SAXS profiles monitored at several time points in the process of the formation of prefibrillar aggregates. The slope of the *I*(*q*) against *q* exhibited a value close to −1, suggesting that prefibrillar aggregates have a rod-like shape. Although this result apparently contradicts the AFM measurements where granular particles were observed (Figure 1D), this is presumably because prefibrillar aggregates are fragile and unstable against washing of the sample plate when preparing the AFM sample [22].

Under an assumption that the shape of the peptide aggregates can be approximately regarded as a rod, we could evaluate the cross-section inertia radius (*R*_c_) from the slope of a cross-section plot. The slope of the cross-section plot was almost constant (Figure 6B) and the time dependence of *R*_c_ suggested that the intermediates maintain the same radius during the measurement time period. The radius of the rod (*R*_p_), which is equal to √2*R*_c_ (see Equation (5)), was calculated to be 3.9 ± 0.3 nm (Figure 6C), and no significant difference was found from that at pH 8.7 estimated in our previous study (3.7 ± 0.1 nm) within experimental errors [24]. By using diffusion coefficient *D*_T_ obtained from DLS and *R*_p_, we further attempted to estimate the length (*L*) of the rod through Equation (6). The *L* values (Figure 6C) showed time-dependent elongation of the rod towards the length of 610 nm, which was longer than the value at pH 8.7 (480 nm) [24]; however, the lengths have relatively large errors and distribution as suggested by DLS (Figure S4), and the approximate shape appeared to be similar, between pH 5.2 and pH 8.7.

## 3. Discussion

The insulin B chain has been shown to have an ability to form amyloid fibrils under a wide range of pH conditions with the help of agitation. In all of the amyloid formations investigated, prefibrillar aggregates were observed immediately after the reaction started, and had a common tendency to accumulate under quiescent conditions as a metastable state. The structure of prefibrillar aggregates was not completely disordered but contained some amount of β-sheet, which is validated by the fact that ThT fluorescence intensity of these aggregates showed slightly positive values. Furthermore, they transformed into amyloid fibrils when subjected to agitation, which would provide mechanical stimuli to prefibrillar aggregates. This behavior is similar to that of prefibrillar aggregates at pH 8.7, which have been suggested to function as a nucleation precursor. It has therefore been suggested that the B chain has a strong propensity for nucleation in a multistep way under a wide range of reaction conditions.

**Figure 7:**
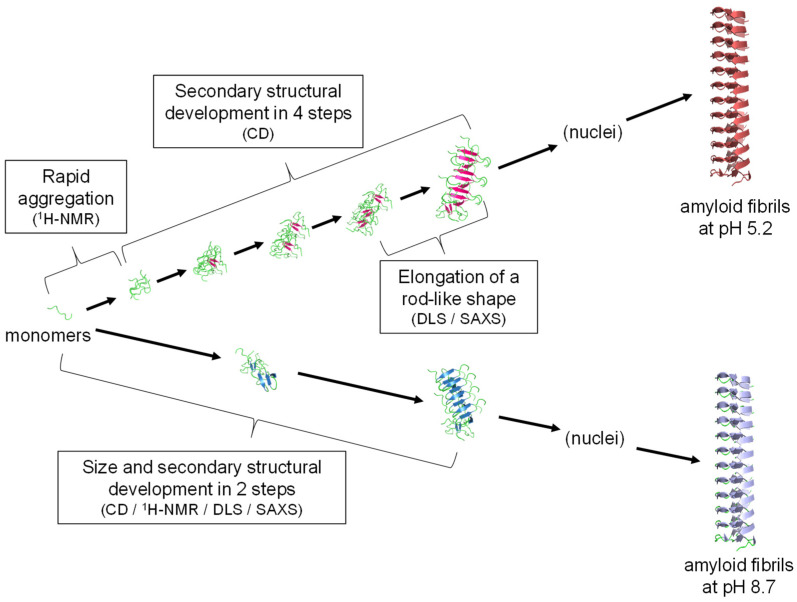
Schematic illustration representing prefibrillar aggregate-mediated amyloid formation of B chain. Two different pathways observed at pH 5.2 and pH 8.7 are shown at the top and bottom of this figure, respectively.

From the FTIR spectra along with proteinase K digestibility and cytotoxicity, structural differences have been revealed between amyloid fibrils formed at pH 5.2 and at pH 8.7. The differences in the final product indicate that the formation pathway of amyloid fibrils varies with pH, which has allowed us to examine prefibrillar aggregates in different pathways. Figure 7 summarizes the reaction schemes proposed at pH 5.2 and pH 8.7. As a basic property of prefibrillar aggregates, rod-like shape and immature β-sheet structure analogous to protofibrils have been revealed irrespective of pathways. In addition, the elongation of the protofibril-like aggregates along the long axis was observed similar to pH 8.7, although the observation was limited at pH 5.2 because the reaction rate was too rapid to be fully tracked. 

While the basic property of prefibrillar aggregates was similar between pH 5.2 and pH 8.7, there were differences in detailed processes. The most notable was the number of steps, with a larger number of intermediates observed at pH 5.2 than at pH 8.7. It was additionally demonstrated that the clustering of peptide molecules preceded their structural development at pH 5.2, unlike pH 8.7, where size and structural development occurred synchronously. The factor that contributes to the difference in the formation process between pH 5.2 and pH 8.7 is considered to be the charged state of the B chain peptide. Given that the p*K*_a_ values of histidine residues at the 5th and the 10th positions, and cysteine residues at the 7th and 19th positions are between these two pHs, these residues are deduced to influence the modes of aggregation. Although only slight differences in size and structure of prefibrillar aggregates could be detected from the analytical techniques used in this work, the pH-dependent change in charge distribution is predicted to modify regions where intermolecular interactions are likely to occur and then to guide to different pathways reaching distinct prefibrillar aggregates.

The tracking of prefibrillar aggregation in the B chain prior to amyloid formation in a comparative way of the two different pathways has shed light on multistep nucleation mechanism of amyloid fibrils. The simplest scheme describing amyloid nucleation is a one-step without intermediates, and in this case, a classical nucleation theory established in crystallography provides an important framework for explaining it reasonably from an energetic view [25,26]. According to this theory, energy change upon crystal formation is described as the sum of the bulk free energy and the interfacial free energy as positive and negative driving forces, respectively. The change in balance of these two energy terms as a function of crystal size and shape produces energy cost until the crystal reaches a critical size, which corresponds to an energy required for nucleation.

In the case of the B chain, however, the nucleation does not fit the classical nucleation theory, and instead, prefibrillar aggregation progresses transiently at an initial stage. The formation of prefibrillar aggregates prior to the appearance of amyloid fibrils is presumed to be due to their kinetic stability. The less organized structure of prefibrillar aggregates tends to exhibit low interfacial energy cost, and therefore the initial energy required for their formation is expected to be low. However, since prefibrillar aggregates are less thermodynamically stable, they need to grow into a more energetically stable state, and eventually, into the most stable amyloid fibril state. Given that the formation of oligomers and protofibrils have been widely observed in many proteins [27], the gradual structural development of prefibrillar aggregates towards amyloid fibrils as observed in the B chain may be one of the representative schemes of amyloid nucleation.

In the multistep nucleation scheme, the process of transforming prefibrillar aggregates to amyloid fibrils is considered the rate-limiting step. It is estimated that direct structural conversion, as proposed in the NCC model, is a candidate mechanism for the structural transformation. Indeed, although not very numerous, some experiments have shown that prefibrillar aggregates convert directly into amyloid fibrils without dissociating into monomers [15,17,28]. Furthermore, computational simulation studies have identified a conversion pathway between prefibrillar and fibrillar forms [29,30]. In addition to this, secondary nucleation, which has recently attracted much attention as an important reaction to proliferate amyloid fibrils [11,12,31], is predicted an alternate mechanism. In this case, the surface of prefibrillar aggregates is conceived to function as a reaction field for secondary nucleation, and prefibrillar aggregates themselves then eventually dissociate to supply peptides for the growth of more stable amyloid fibrils. Although it is not possible to conclude exactly the mechanism actually adopted, direct conversion might be a strong candidate considering that amyloid formation occurred via prefibrillar aggregates even at pH 5.2, where almost all fraction of the monomers was depleted after the formation of prefibrillar aggregates. 

The previous observation at pH 8.7 has suggested that specific structural features of prefibrillar aggregates play an important role when they mediate nucleation. Prefibrillar aggregates are predicted to link to amyloid fibrils on the energy landscape when their structure allows the transition energy to amyloid fibrils to be reduced enough to be overcome. Interestingly, FTIR spectra commonly showed that prefibrillar aggregates of the B chain contained a small amount of antiparallel β-sheet structures, as seen in many other oligomers and protofibrils [29,32,33,34], although the significance of such a structural property for the transition to the fibril state is still unknown. Given that similar structural properties are observed regardless of pH conditions, the nonfibrillar aggregates at other pH conditions are also expected to convert to amyloid fibrils, as at pH 8.7 and 5.2. However, nucleation from coexisting monomers may also need to be considered, especially at pH 9.1 where only a small amount of aggregates is formed. Clarifying detailed structures of various prefibrillar aggregates, as well as capturing the moment when they form nuclei, will reveal the exact role of prefibrillar aggregates.

Furthermore, the identified variety in evolution of prefibrillar aggregates has provided insights into amyloid polymorphism. Diverse amyloid structures formed from the same protein or polypeptide have recently attracted attention since they have been proposed to be involved in different pathologies [35]. The present study has suggested that different patterns of prefibrillar aggregation accompany polymorphic pathways. Prefibrillar aggregation is estimated to proceed under conditions that exceed the peptide solubility limit, and where the peptides form a variety of assembly structures reflecting the state of the peptides. The resulting diversity of prefibrillar aggregates is expected to guide various pathways for amyloid formation. Investigating amyloid formation pathways in terms of prefibrillar aggregates will provide deeper understanding not only of nucleation itself, but also of pathogenesis associated with polymorphism.

## 4. Materials and Methods

### 4.1. Purification of Insulin B Chain

Insulin B chain was isolated from human insulin (FUJIFILM Wako Pure Chemical, Osaka, Japan) as in our previous works [21,22]. The B chain was dissolved in 10 mM NaOH and stocked at −80 °C before use. The purity of the B chain was assessed by the ^1^H signals of ε protons in tyrosine residues obtained using the NMR spectrometer, AVANCEIII HD (Bruker, Billerica, MA, USA). The concentration of B chain was determined by using the absorption coefficient of 0.90 (mg/mL)^−1^cm^−1^ at 280 nm in 10 mM NaOH solution.

### 4.2. Formation of B Chain Amyloid Fibrils

The formation of amyloid fibrils of B chain was carried out with peptide concentration ranging from 0.22 to 1.40 mg/mL (i.e., 64 to 408 μM) and at pH ranging from 3.0 to 9.1. The B chain in 10 mM NaOH was diluted by an approximately half volume of an appropriate buffer, i.e., glycine for pH 3.0, acetate for pH 5.2, phosphate for pH 6.4, 7.0, and 7.8, and Tris for pH 8.7 and 9.1, to reach a final peptide concentration. The final buffer concentration was 50 mM, and the sample contained 5 mM NaCl generated by neutralizing NaOH in the B chain solution. All experiments were performed at 25 °C under agitated or quiescent conditions. In experiments conducted under agitated conditions, the samples in microtubes were incubated under continuous shaking at 1200 rpm using a ThermoMixer C (Eppendorf, Hamburg, Germany). In experiments conducted under quiescent conditions, samples prepared in microtubes were placed in an air incubator.

### 4.3. ThT Assay

The formation of amyloid fibrils was monitored by ThT fluorescence. In the assay, 5 μL of a sample solution was mixed with 1.5 mL of a ThT assay solution, which was composed of 5 μM ThT and 50 mM glycine buffer (pH 8.5). One minute after the incubation, fluorescent intensity was recorded at 485 nm with an excitation at 445 nm using the spectrofluorometer, RF-5300pc (Shimadzu, Kyoto, Japan).

### 4.4. AFM

AFM images were obtained using micro cantilever (OLYMPUS, Tokyo, Japan) and the dynamic force mode with Probestation NanoNavi II/IIe (Hitachi High-Tech Science, Tokyo, Japan). Five to twenty microliters of a sample were loaded to a mica plate, left for one minute, and then rinsed using 200 μL of water. The sweep rate was set to 0.5 or 1.0 Hz.

### 4.5. ATR-FTIR Spectroscopy

ATR-FTIR spectra were measured with a Nicolet iS5 FT-IR equipped with an iD5 ATR accessory (Thermo Fisher Scientific, Waltham, MA, USA). Samples of all pH conditions were precipitated by centrifugation in the presence of 0.5 M NaCl and resuspended in water. This process was repeated three times to replace the buffer solution in the sample with water. The suspension was placed on a diamond crystal prism and then dried. FTIR measurement was performed by collecting 128 interferograms at a resolution of 4 cm^−1^.

### 4.6. CD Spectroscopy

Far-UV CD spectra were measured to investigate the secondary structure of the product, as well as to track the time-dependent structural changes. CD spectra were obtained using a CD spectrometer, J-1100 (JASCO, Tokyo, Japan). A sample was placed in a quartz cell with a path length of 0.2 mm or 1.0 mm. Each scan was performed at 200 nm/min, and eight individual scans were averaged to obtain one spectrum. A time-course plot obtained under quiescent conditions was fitted using an exponential or a biexponential equation below:

(1) $$\left[\theta \right]\left(t\right)={\left[\theta \right]}_{1}-({\left[\theta \right]}_{1}-\left[\theta {]}_{0}\right)\;\exp\left(-\frac{t}{\tau_{1}}\right)$$

(2) $$\left[\theta \right]\left(t\right)={\left[\theta \right]}_{2}-\left([\theta {]}_{1}-[\theta {]}_{0}\right)\exp\left(-\frac{t}{\tau_{1}}\right)-\left([\theta {]}_{2}-[\theta {]}_{1}\right)\exp\left(-\frac{t}{\tau_{2}}\right)$$

where *τ_i_* and [*θ*]*_i_* represent the apparent time constants of the *i*th phase and the asymptotic value of mean residue molar ellipticity after the completion of the *i*th phase, respectively. For fitting, the time dependence of the [*θ*] values at intervals of 1 nm over the wavelength range of 200–250 nm at 0.22 and 1.40 mg/mL, 205–250 nm at 0.25 and 0.30 mg/mL, or 215–250 nm at 0.50 and 0.70 mg/mL was used.

### 4.7. ^1^H-NMR Spectroscopy

The monomer concentration was estimated from the decrease of the NMR spectrum signal. A sample was placed in a glass tube with an inner diameter of 5 mm and incubated at 25 °C. NMR spectra were measured using AVANCE III HD with a superconducting magnet with a Larmor frequency of 400.13 MHz (Bruker, Germany). The spectrometer was controlled using the programs of Topspin 1.5 and IconNMR (Bruker). High homogeneity of the magnetic field was achieved by Topspim, a routine tool built by Topspin 1.5, and a pulse program, zgesgp, was used for spectral measurements.

### 4.8. SAXS

SAXS measurements were performed by NANOPIX equipped with a HyPix-6000 (Rigaku, Tokyo, Japan). A Cu K-α line (MicroMAX-007HF; Rigaku) was used as a beam source and the camera length was set to 1.33 m. A sample liquid was loaded into a made-to-order sample cell consisting of a pair of quartz windows having an optical path length of 1 mm, incubated under quiescent conditions at 25 °C, and time-lapse measurements were performed by continuous data acquisition with an exposure time of 15 min for each profile. The magnitude of scattering vector *q* (*q* = 4πsin(*θ*/2)/*λ*, where *λ* and *θ* indicate X-ray wavelength and the scatter angle, respectively) ranged from 0.0085 to 0.2 Å^−1^. The Porod region of the double logarithm plots of the SAXS profiles was analyzed to evaluate the approximate shape of aggregates using the equation below [36]:

(3) logI(q)=logI(0)+a·log(q)

where *I*(0) and a represent the intensity at *q* = 0 and the slope of the double logarithm plot, respectively. In the analysis of aggregates with a rod-like shape, cross-section plots were constructed and fitted using the equation below [37]:

(4) $$\log\left\{I\left(q\right)\cdot q\right\}=A-\frac{R_{c}^{2}}{2}q^{2}$$

where *A* represents the constant, and *R*_c_ represents the radius of the cross-section. The maximum *q* value for the fitting region is restricted to *R*_c_ *q* < 1.3. The radius of a rod, i.e., *R*_p_, is described as

(5) $$R_{p}=\sqrt{2}R_{c}$$

The length of prefibrillar aggregate, *L*, is obtained by Broersma’s relationship [38]:

(6) $$D_{T}=\frac{k_{B}T}{3\pi \eta_{0}L}\ln\left(\frac{L}{R_{p}}\right)$$

where *D*_T_ is the diffusion coefficient obtained by DLS analyses (see Supplementary Materials), and *k*_B_, *T*, and *η*_0_ represent the Boltzmann constant, temperature, and viscosity, respectively.

## Data Availability

Data are contained within the article or Supplementary Materials.

## Supplementary Materials

The following supporting information can be downloaded at: https://www.mdpi.com/article/10.3390/molecules27133964/s1, Supplementary Methods: Proteinase K digestion of amyloid fibrils; Cytotoxicity assay; DLS. Figure S1: Proteinase K digestion of amyloid fibrils formed at pH 5.2 and pH 8.7.; Figure S2: Cell viability against the B chain amyloid fibrils formed at pH 5.2 and pH 8.7.; Figure S3: CD spectra monitored in the formation of amyloid fibrils under agitated conditions at pH 5.2.; Figure S4: Time-dependent changes in hydrodynamic diameter during the formation of prefibrillar aggregates at pH 5.2. Ref. [39] cited in Supplementary Materials.

## Sample Availability

There are no samples of the compounds that are available from the authors.
