# Supplementary material for: Pathway Dependence of the Formation and Development of Prefibrillar Aggregates in Insulin B Chain
Source: Molecules. 2022 Jun 21;27(13):3964. doi: 10.3390/molecules27133964 (PMC9268647; doi:10.3390/molecules27133964)
Supplement: Supplementary file 1 [file molecules-27-03964-s001.zip › molecules-1771871-supplementary.pdf]

Supplementary Materials

Pathway dependence of the formation and development of prefibrillar aggregates in insulin B chain

Yuki Yoshikawa[1], Keisuke Yuzu[1], Naoki Yamamoto[2], Ken Morishima[3], Rintaro Inoue[3], Masaaki Sugiyama[3], Tetsushi Iwasaki[1,4], Masatomo So[5], Yuji Goto[6], Atsuo Tamura[1], and Eri Chatani[1]

[1]Graduate School of Science, Kobe University, 1-1 Rokkodai, Nada, Kobe, Hyogo 657-8501, Japan

[2]Division of Biophysics, Physiology, School of Medicine, Jichi Medical University, 3311-1 Yakushiji, Shimotsuke, Tochigi 329-0498, Japan

[3]Institute for Integrated Radiation and Nuclear Science, Kyoto University, 2 Asashiro-Nishi, Kumatori, Sennan-gun, Osaka 590-0494, Japan

[4]Biosignal Research Center, Kobe University, 1-1 Rokkodai, Nada, Kobe, Hyogo 657-8501, Japan

[5]Institute for Protein Research, Osaka University, 3-2 Yamadaoka, Suita, Osaka 565-0871, Japan

[6]Global Center for Medical Engineering and Informatics, Osaka University, 2-1 Yamadaoka, Suita, Osaka 565-0871, Japan

Supplementary Methods

Proteinase K digestion of amyloid fibrils. Amyloid fibrils of B chain were formed at a peptide concentration of 1.40 mg/ml, and then they were collected by centrifugation as a precipitant. After the supernatant was removed, the pellet was suspended with the same volume of 50 mM Tris-HCl (pH 7.5) containing 0.15 mg/ml Proteinase K (Nacalai Tesque, Kyoto, Japan), and proteinase digestion was conducted for 15 min under shaking conditions at 600 rpm and 25 °C with a ThermoMixer C (Eppendorf, Hamburg, Germany). After the reaction, the sample was centrifugated again, and the pellet was then dissolved by adding the same volume of 75% dimethyl sulfoxide in 50 mM Tris-HCl (pH 7.5). The peptide fragments generated were subjected to liquid chromatography mass spectrometry (LC-MS) analysis with an LCQ Fleet (Thermo Fisher Scientific, Waltham, MA, USA). Twenty microliters of the sample was injected, and the chromatographic separation was performed on a column 5C18-AR-II (20 mm I.D.×50 mm). The mobile phase consisted of 0.1% trifluoroacetic acid (A) and acetonitrile with 0.1% trifluoroacetic acid (B). The flow rate was 0.5 ml/min, with a linear gradient of B at 1%/ml from 0 to 35%.

Cytotoxicity assay. Cellular toxicity was evaluated by quantifying cell viability using Crystal Violet staining as described in a previous study [1]. PC12 cells were cultured in Dulbecco's Modified Eagle's medium containing 10% fetal bovine serum, 5% horse serum, 100 U/ml penicillin G, and 100 μg/ml streptomycin a humidified atmosphere of 5% CO_2 at 37 °C. The cells were dispensed into 96-well glass bottom plates at an approximate number of 1×10^4 cells/well and incubated for a day. Differentiation was induced by adding nerve growth factor-β to a final concentration of 10 ng/ml. The cells were then treated with amyloid fibrils at various concentrations for a day. After the treatment, 100 μl of 3% formaldehyde solution in phosphate buffered saline was added to immobilize cells on the plate. Staining was performed by adding 100 μl of 0.1% Crystal Violet solubilized in 10% ethanol and incubating for an hour. The stained

plate was washed gently with water, dried, and scanned, and the degree of staining was quantified by an Image J. The cell viability was calculated from the ratio of the values to the control.

Dynamic light scattering (DLS). DLS measurements were performed using Zetasizer Nano-S (Malvern Panalytical, Worcestershire, UK). A He-Ne laser with a wavelength of 633 nm was irradiated to a sample placed in an Ultra-Micro cell (Hellma, Müllheim, Germany), and backward scattered light was detected using an avalanche photodiode with a scattering angle of 173°. Data collection and processing were performed using the software, Dispersion Technology Software 5.00 (Malvern Panalytical). Size distribution was obtained using the non-negative least-squares (NNLS) method, in which hydrodynamic diameter D_h is described by the Stokes-Einstein relationship:

$$D_\mathrm{h} = \frac{k_\mathrm{B}T}{3\pi\eta D_\mathrm{T}} \qquad \text{(S1)}$$

where T, η, and D_T represent the temperature, viscosity of the system, and diffusion coefficient, respectively. The parameters: $n = 1.331$, $\lambda_0 = 633$ nm, $\theta = 173°$, $k_B = 1.386 \times 10^{-23}$ J/K, $\eta = 8.93 \times 10^{-4}$ Pa·s, and $T = 298.15$ K were used for the calculation. The diffusion coefficient D_T was further used for the calculation of the length of the prefibrillar aggregates (L) with an assumption that they have a rod-like shape (see eq. 6 and Figure 6C in the main text).

Reference

1. Nagano, T.; Iwasaki, T.; Onishi, K.; Awai, Y.; Terachi, A.; Kuwaba, S.; Asano, S.; Katasho, R.; Nagai, K.; Nakashima, A.; Kikkawa, U.; Kamada, S. Ly6d-induced macropinocytosis as a survival mechanism of senescent cells, *J Biol Chem.* **2021**, 296, 100049.

Supplementary Results

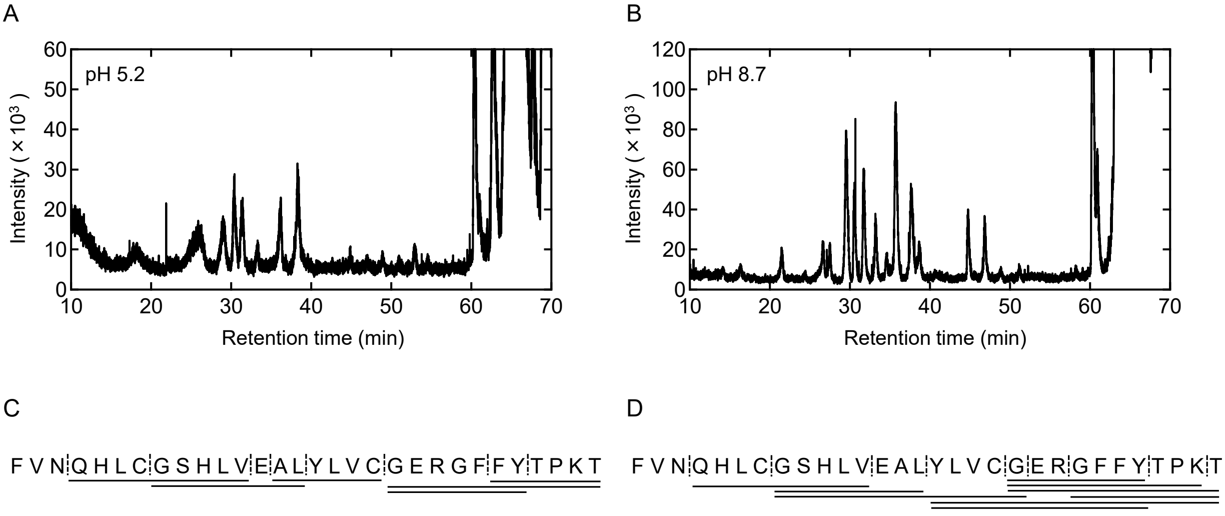

Figure S1. Proteinase K digestion of amyloid fibrils formed at pH 5.2 and pH 8.7. Peptide fragments formed by the proteinase K digestion of amyloid fibrils were analyzed by LC-MS. (**A**,**B**) Total ion chromatograms of the proteinase K-digested amyloid fibrils formed at pH 5.2 (A) and pH 8.7 (B). (**C**,**D**) Summaries of identified peptides at pH 5.2 (C) and pH 8.7 (D). The identified peptides are represented by solid lines below the amino acid sequence of the B chain. Broken lines are cleavage sites estimated from the identified peptides.

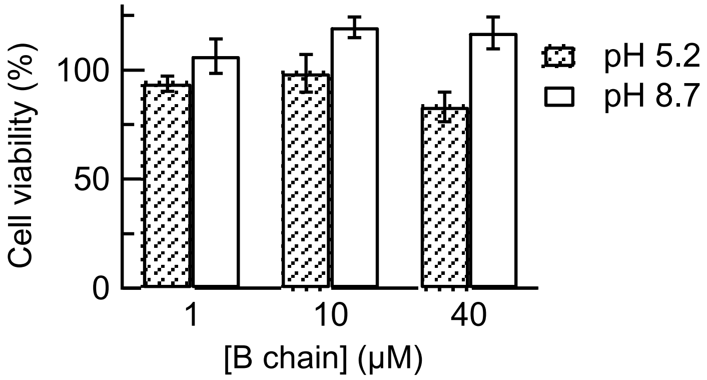

Figure S2. Cell viability against the B chain amyloid fibrils formed at pH 5.2 and pH 8.7. Cellular toxicity of amyloid fibrils of the B chain was evaluated by Crystal Violet staining assay using PC12 cells. Results at three different concentrations of amyloid fibrils, 1, 10, 40 μM (in monomeric unit; i.e., 3.4×10^{-3}, 3.4×10^{-2}, and 1.4×10^{-1} mg/ml, respectively) are shown. Each bar represents the mean and standard deviation of triple measurements.

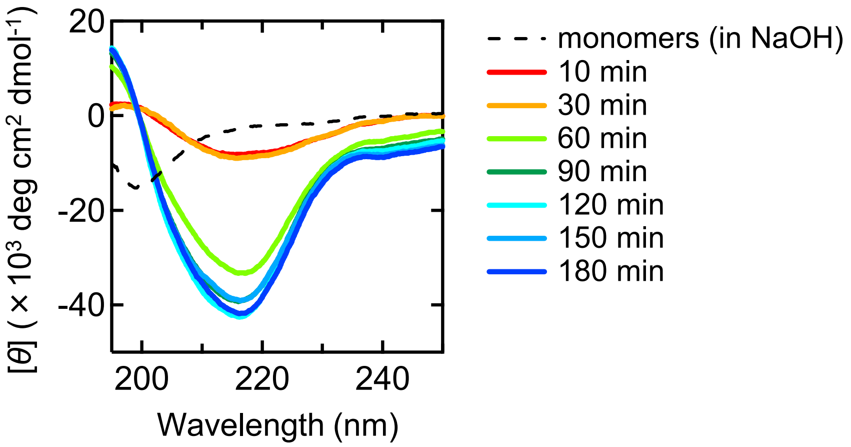

Figure S3. CD spectra monitored in the formation of amyloid fibrils under agitated conditions at pH 5.2. The formation of amyloid fibrils of the B chain was performed at 1.40 mg/ml and 25 °C, and during the reaction, an aliquot of the reaction mixture was taken at different time point and subjected to the CD measurement. The spectrum changed in two steps, suggesting that prefibrillar aggregates were formed immediately after the start of the reaction, which was followed by the formation of amyloid fibrils after accumulating for some time period, suggesting that prefibrillar aggregates are initially formed and then transformed into amyloid fibrils.

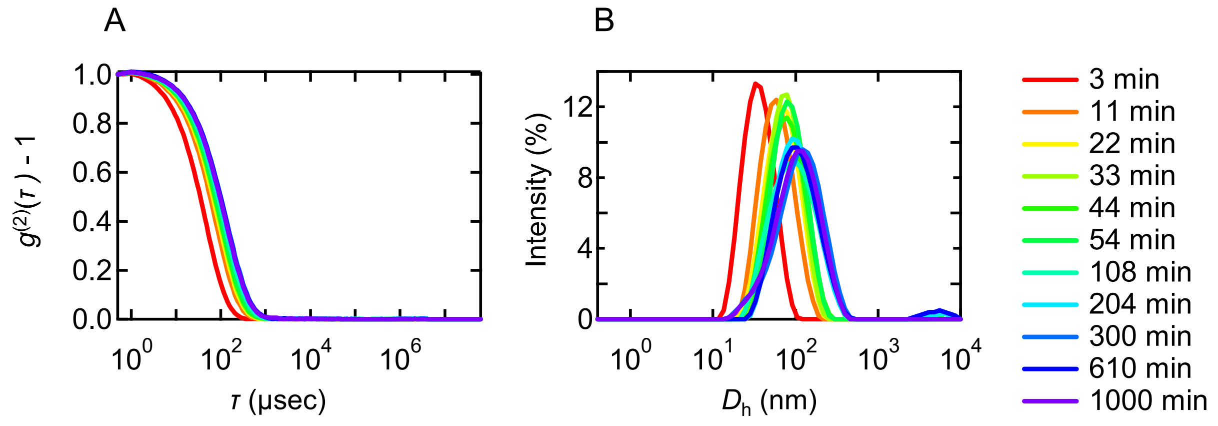

Figure S4. Time-dependent changes in hydrodynamic diameter during the formation of prefibrillar aggregates at pH 5.2. (**A**) Time changes in autocorrelation function $g^{(2)}(\tau)-1$. Normalized traces are shown in this figure. (**B**) Size distributions obtained from the autocorrelation functions. The formation of prefibrillar aggregates of the B chain was observed at 1.40 mg/ml and 25 °C. With the D_h value at the peak top of each distribution and the R_p value obtained from the SAXS measurement, the length of prefibrillar aggregate L was estimated using eq. S1 and 6, as shown in Figure 6C.
